# Fine Needle Aspiration Cytology in Unveiling a Rare Diagnosis of Lacrimal Sac Solitary Fibrous Tumour and Its Cytological Attributes

**DOI:** 10.7759/cureus.102161

**Published:** 2026-01-23

**Authors:** Siddhartha Banchhor, Vaishali Walke, Deepti Joshi, Vikas Gupta

**Affiliations:** 1 Pathology and Laboratory Medicine, All India Institute of Medical Sciences, Bhopal, Bhopal, IND; 2 Otorhinolaryngology, All India Institute of Medical Sciences, Bhopal, Bhopal, IND

**Keywords:** immunocytochemistry, lacrimal sac tumour, ocular pathology, solitary fibrous tumour, stat6

## Abstract

Solitary fibrous tumour (SFT) of the lacrimal sac is an uncommon lesion that may present as a slow-growing, painless medial canthal swelling, and its cytological features are not widely characterised. We report the case of a 48-year-old male patient who presented with a firm, non-tender 4 × 4 cm mass at the left medial canthus with gradual progression over several months. Fine-needle aspiration smears were moderately cellular and showed spindle to oval cells arranged in loose clusters, singly scattered, and focally adherent to endothelial fragments. The cells exhibited pale wispy cytoplasm with elongated processes, round to oval nuclei with fine chromatin and inconspicuous nucleoli, without necrosis or significant atypia. Immunocytochemistry performed on the destained smears demonstrated a diffuse cluster of differentiation (CD)34 positivity. Radiological evaluation revealed a well-circumscribed, enhancing lesion expanding the lacrimal sac and nasolacrimal duct. Complete excision of the lesion was undertaken. Histopathology showed a cellular spindle cell tumour arranged in short fascicles and whorls with a prominent branching vascular network. Immunohistochemistry confirmed the diagnosis with diffuse cytoplasmic CD34 and strong nuclear Signal Transducer and Activator of Transcription 6 (STAT6) expression. No significant mitotic activity or necrosis was identified.

## Introduction

First described by Klemperer and Rabin in 1931, Solitary Fibrous Tumour (SFT) is now recognised as a NGFI-A Binding Protein 2‑Signal Transducer and Activator of Transcription 6 (NAB2-STAT6) fusion-driven neoplasm, with the resulting nuclear STAT6 immunoreactivity, which is a valuable diagnostic clue [1]. Although the vast majority of these tumours behave indolently, about 10-15% exhibit local recurrence or late metastasis. Thus, underscoring the importance of precise pre‑operative identification, especially in functionally critical anatomical locations such as the lacrimal drainage system [2]. Fewer than a dozen well‑documented cases involving the lacrimal sac have been reported in the literature; most of the cases quoted had been diagnosed only after surgery, and the cytological descriptions are available in only a few reports[3]. On fine‑needle aspiration, the tumour can mimic spindle cell tumours such as cellular schwannoma, hemangiopericytoma‑like vascular tumors or dermatofibrosarcoma, making clinico‑radiological correlation and judicious use of immunohistochemistry (IHC) markers an essential step for substantiating the diagnosis. Preoperative diagnosis facilitates the complete, margin‑negative but vital structure‑preserving surgical procedures that can further help avoid both overtreatment and recurrence.

## Case presentation

**Figure 1:**
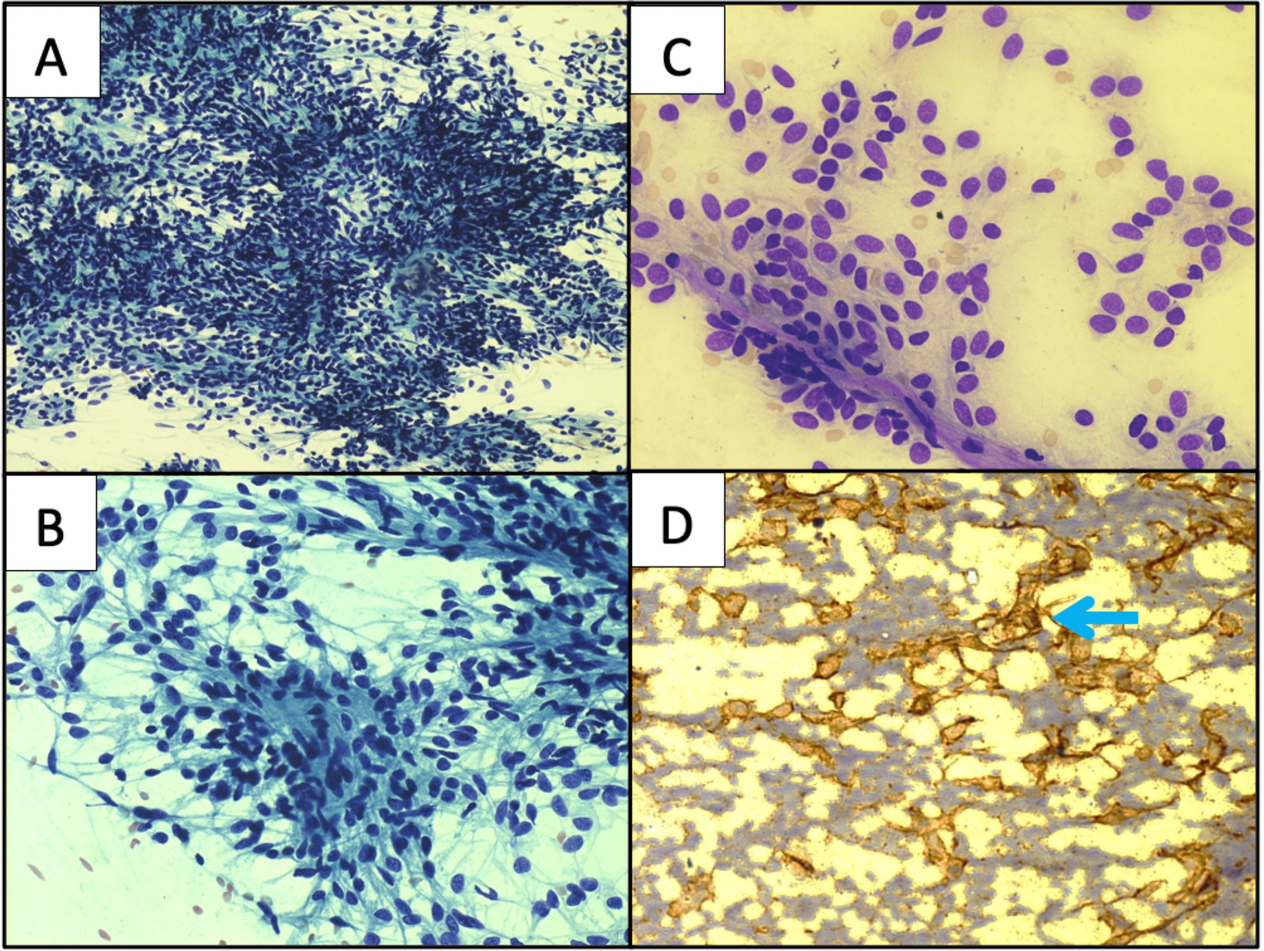
Cytology of the fine needle aspiration (FNA) smears A & B: Tumour cells in loose groups and singly scattered of spindle shaped displaying pale wispy cytoplasm, round to oval nuclei and regular nuclear membrane, fine uniform chromatin and inconspicuous nucleoli (Papanicolaou (PAP); 10X (A) and 40X (B)); C: Cohesive clusters of tumour cells seen adherent to branching endothelial fragments (Giemsa; 20X); D: Granular cytoplasmic positivity evident in tumour cells (Immunohistochemistry (IHC) cluster of differentiation (CD)34; 20X).

A 48-year-old male patient presented to the cytology clinic with a history of swelling over the left medial canthus for about a period of seven months. It was painless and gradually progressive. On local examination, it measured 4 x 4 cm, was single, firm, non-tender, and mobile. Understanding the superficial nature of the lesion, fine needle aspiration cytology (FNAC) was tried using 24G needle by the non-aspiration technique after obtaining consent and explaining the procedure to the patient. The smears prepared were immediately wet fixed in 90% ethanol and air dried; later stained with Papanicolaou (PAP) and Giemsa stain. The cellular smears revealed a predominant population of spindled to oval cells in loose clusters, as well as adherent to endothelial capillary fragments and also singly scattered. These cells displayed scant to moderate amounts of pale, wispy cytoplasm with elongated cytoplasmic processes. The nuclei were round to oval, having pale, uniform chromatin and inconspicuous nucleoli. There was no evidence of any anaplasia, mitosis or necrosis observed in the smears examined (Figures 1A-1C).

**Figure 2:**
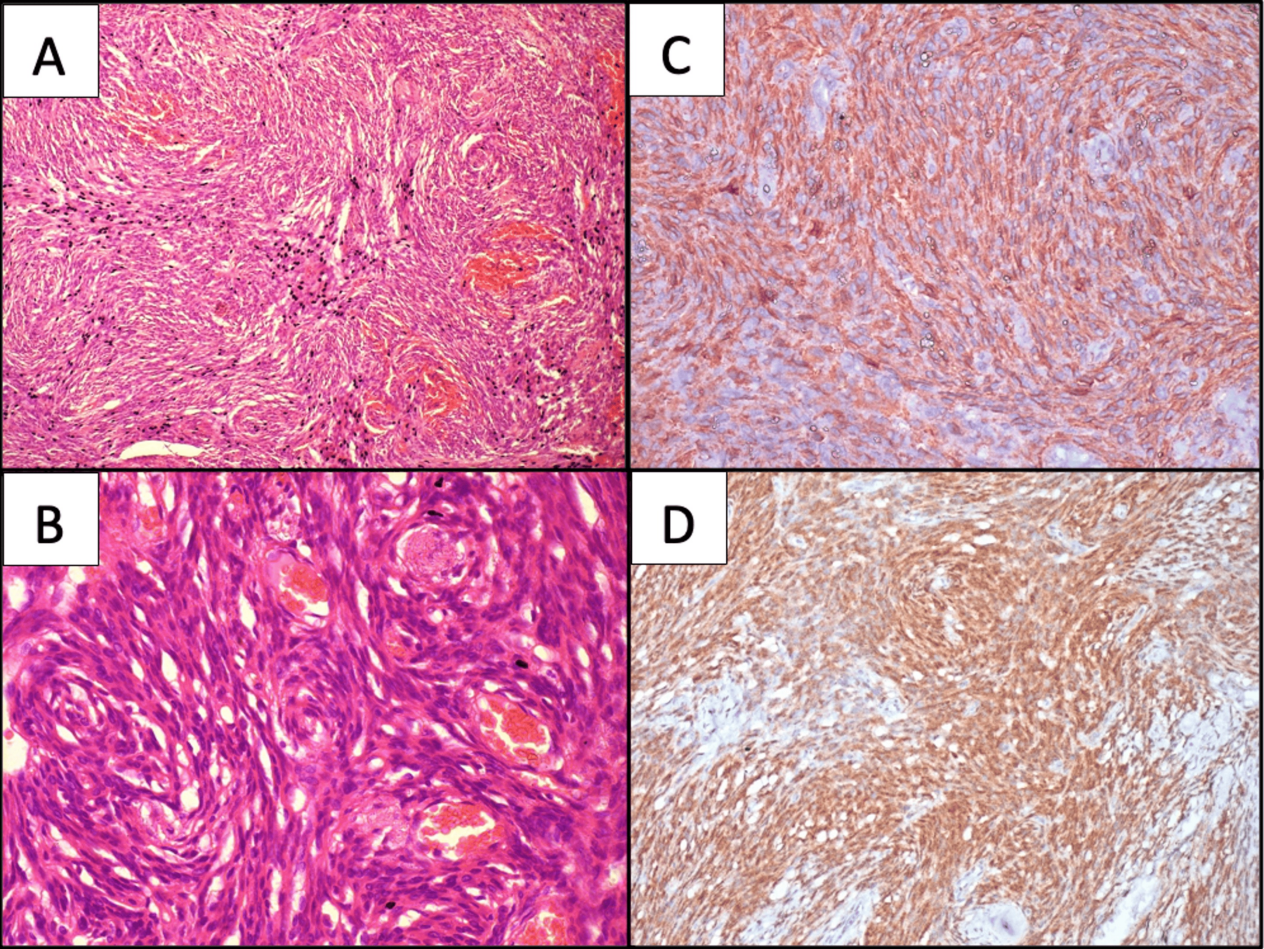
Histopathology & immunohistochemistry (IHC) A&B: Cellular spindle cell tumour shows cells in whorls, short fascicles and storiform pattern at places. The cells have indistinct cellular borders, plump oval to spindle-shaped nuclei, fine chromatin, inconspicuous nucleoli and a moderate amount of pale cytoplasm. Mitotic activity is inconspicuous. Numerous small to variable-sized blood vessels also noted within the tumour proper (Hematoxylin and Eosin (H&E); 10X (A) and 20X (B)); C: Tumour cells show diffuse granular cytoplasmic positivity (IHC CD34; 20X); D: With STAT6, diffuse strong nuclear positivity in tumour cells (IHC STAT6; 20X)

Meanwhile radiological findings were available which revealed a well-circumscribed, homogeneously enhancing iso-dense mass in medial canthus, causing widening of ipsilateral lacrimal sac and nasolacrimal duct. Radiological suspicion was for lymphoma (Figure 2).

**Figure 3:**
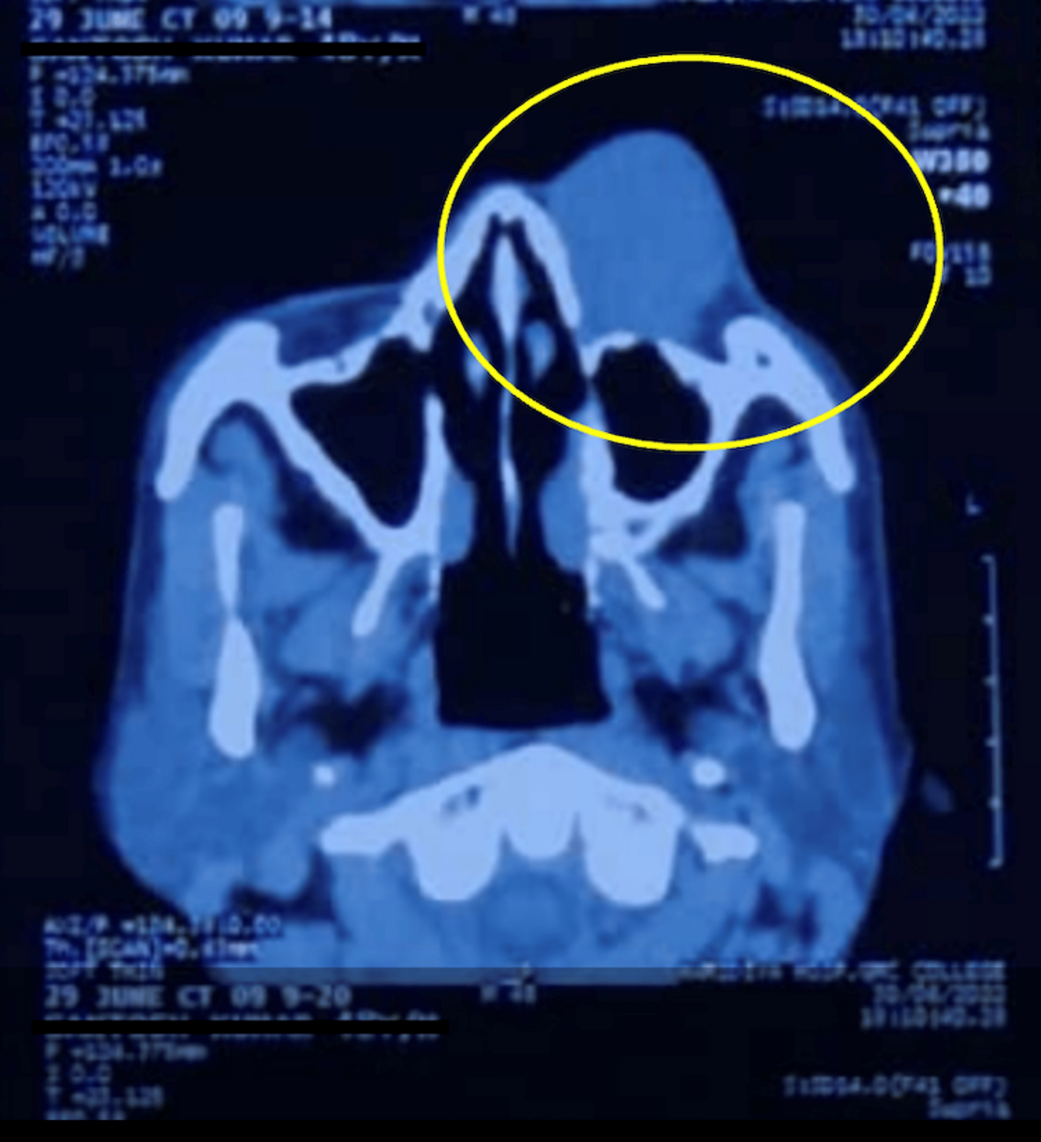
Axial section of the non-contrast CT orbit CT scan image with axial view of the head shows a well-defined, round to oval, homogeneous soft-tissue lesion is noted in the left medial canthal and lacrimal sac region. The yellow oval demarcation shows smooth outward bulging of the overlying skin. The lesion produces mild pressure on the medial side of the left eyeball from the superolateral aspect, without any evidence of enophthalmos, exophthalmos, or intraconal extension. Medially, there is mild extension into the proximal left nasolacrimal duct with associated bony expansion.

Based on the cytomorphological features and imaging findings, the possibility of spindle cell neoplasm favoring SFT was offered. Immunocytochemistry (ICC) for CD34 performed on destained Giemsa smears, showed diffuse cytoplasmic positivity in most of the tumour cells (Figure 1D). Subsequently the tumour was excised and a resection specimen received for histopathology. The tumor measured 4 x 3 x 3 cm, was well encapsulated and firm. The cut surface of the tumour displayed solid, homogenous focal with areas of haemorrhages. The multiple sections examined revealed the tumour focally covered by stratified columnar epithelium with presence of goblet cells. The subepithelial tissue showed the presence of a cellular spindle cell neoplasm forming whorls, short fascicles and storiform pattern at places. These cells had indistinct cellular borders, plump oval to spindle-shaped nuclei, fine chromatin, inconspicuous nucleoli, and a moderate amount of cytoplasm. Mitotic activity was inconspicuous. Intervening stroma showed numerous small to variable sized blood vessels. Focal areas of haemorrhage were also noted. Areas of necrosis were not observed (Figure 3).

The panel of IHC markers applied revealed CD34 with diffuse cytoplasmic positivity, while smooth muscle actin (SMA), soluble protein 100 (S100), and desmin were negative. STAT6 was performed as the advanced panel revealed a strong diffuse nuclear positivity in tumour cells. The cells however did not express CD99. In view of the histomorphology and panel of immunomarkers, a final diagnosis of SFT was tendered.

## Discussion

SFT of the lacrimal passage remains an exceptionally rare neoplasm [3]. Earlier series enumerated fewer well‑documented cases, most of which had been diagnosed only after the surgical excision, and occasional reports on the cytological descriptions [3,4]. A recent review of a 12-year-old girl with a nasolacrimal‑duct SFT raised the cumulative global total to just 18 patients and confirmed the broad age spectrum (12-79 years; mean ≈ 43) [4]. Against this, the present case involves a 48-year-old male patient with a medial canthal mass who presented with a painless, slowly enlarging swelling. This clinical presentation reflects the indolent behaviour described by Lai et al. [3].

Notably, pediatric involvement was also recognised following Sheth et al.’s case [4]; therefore, age can no longer reliably exclude SFT as one of the differential diagnoses in the peri‑ocular region. Radiologically, all three reports, including the present case, described a sharply circumscribed, homogeneously enhancing lesion that expands, rather than infiltrates, the adjacent lacrimal structures. Such imaging concordance supports pre‑operative consideration of SFT whenever a well‑defined medial‑orbit mass abuts the nasolacrimal apparatus. FNAC in the present case yielded a moderately cellular smear composed of bland spindle‑to‑oval to plump cells, adherent to endothelial fragments. The cells exhibited finely granular, uniform chromatin with inconspicuous nucleoli and lacked necrosis or anaplasia. Samaddar et al. (2021) emphasised that this pattern may mimic cellular schwannoma, low‑grade fibromyxoid sarcoma, and dermatofibrosarcoma, rendering ancillary tests crucial [5].

The diffuse CD34 reactivity on IHC has long been recognised, but the seminal work by Koelsche et al. has established the nuclear relocation of STAT6 as a highly specific surrogate marker for the pathognomonic NAB2‑STAT6 fusion responsible for the definitive diagnosis of SFT [1]. In the present case, clinico-radiological findings, cytological features [6], guided the specific CD34 ICC, followed by histology, and substantiated by STAT6 nuclear expression on IHC. The characteristic presence of staghorn-type vessels may be appreciated on cell blocks and rare findings on cytology. However, the presence of tumour cells adherent to the endothelial fragments, as seen in the current case, may be a reflection of the increased vascularity, as noted on tissue sections [7]. Although these patterns are suggestive, specific cytologic diagnosis is often limited without an ancillary test because of overlapping features of spindle-cell lesions [8-10]. Emerging evidence for p53, p16 and Ki‑67 scoring can be integrated into future lacrimal-sac case series to harmonise outcome reporting [11].

Smears from the schwannoma demonstrated cohesive fascicles of spindle cells with elongated nuclei showing tapering, buckled ends and fine chromatin. Intranuclear inclusion, verocay bodies, and ropy collagen can be additional findings. Strong and diffuse positivity for S100 and SOX10 is characteristic of schwannoma, while STAT6 is negative [8,12,13]. Low-grade myxofibrosarcoma can display spindle cells with minimal nuclear pleomorphism, against a background of myxoid stroma well appreciated in Giemsa, and delicate thin-walled curvilinear blood vessels; these features altogether need to be interpreted in the right clinical context. The single best discriminator is diffuse strong mucin 4 (MUC4) immunoreactivity and absence of STAT6 nuclear staining [5].

Dermatofibrosarcoma can be considered a close morphological differential because of overlapping features, including a bland-appearing spindle cell morphology with a whorled pattern and demonstrating CD34 positivity, mimicking SFT. However, clinic-radiological findings such as the presence of superficial dermal location of the tumour as well as a lack of the staghorn vessel pattern may aid in distinguishing it and this can be further supported by ancillary markers like STAT6 [13]. Monophasic synovial sarcoma is a rare entity for the periocular region. However, it can be mistaken for SFT due to the uniform, monotonous fascicular spindles that may overlap with SFT on FNAC. Although synovial sarcoma commonly shows Transducin-Like Enhancer of Split 1 (TLE1; high sensitivity), many cases express keratin/Epithelial Membrane Antigen (EMA), while STAT6 is negative. Definitive confirmation rests on Synovial Sarcoma Translocation, Chromosome 18 (SS18) rearrangement (break-apart Fluorescence In Situ Hybridization or FISH) or SS18-18-Synovial Sarcoma X breakpoint (SSX) fusion by Reverse Transcription Polymerase Chain Reaction/Next-Generation Sequencing (RT-PCR/NGS) [13]. Adachi et al. showed that cases with cytoplasmic (rather than nuclear‑restricted) STAT6 and high Tumor Protein p53/Cyclin-Dependent Kinase Inhibitor 2A (p53/p16) expression portend a greater likelihood of malignant behaviour in orbital SFTs [14]. Although the current case exhibited unequivocal nuclear STAT6 without aberrant p53/p16) staining, the finding underscores the prudence of incorporating extended IHC panels, particularly in sites like the lacrimal sac, where complete excision may be surgically challenging [14,15]. Long‑term outcome data remain limited.

Liu et al.’s meta‑analysis of pleural SFTs (n=427) reported the cumulative 10‑year recurrence of 11 %, with mitotic index >4/10 high-power field or HPF and tumor size >10 cm as independent risk factors [2]. The lacrimal‑sac series to date are smaller (<20 cases) but align with these predictors in studies by Sheth et al. and Lai et al. The tumours featuring increased mitoses or positive surgical margins have reported to recur within two years despite adjuvant radiotherapy [3,4]. The tumour in the present case measured 3.6 cm in size, displayed negligible mitotic activity, and was excised with negative margins. Nevertheless, regular imaging surveillance is advised, because late relapse (>15 years) is documented for extra‑pleural sites [2].

## Conclusions

SFT of the lacrimal sac represents an exceptionally uncommon diagnostic entity, often posing significant preoperative challenges because of its non-specific clinical presentation and overlap with other spindle cell lesions of the periocular region. As highlighted in the present case, careful assessment of cytomorphological features, namely, moderately cellular smears composed of bland spindle to oval cells with pale, wispy cytoplasm, fine chromatin, inconspicuous nucleoli, and characteristic adherence to thin-walled endothelial fragments, can provide an important initial diagnostic clue. When these cytological findings are interpreted in conjunction with clinico-radiological features and supported by targeted ICC, particularly diffuse CD34 positivity, a confident preoperative suspicion of SFT can be achieved even at rare and anatomically constrained sites such as the lacrimal sac.

The diagnostic certainty is further strengthened by the demonstration of strong and diffuse nuclear STAT6 expression on histopathology, serving as a highly specific surrogate marker for the underlying NAB2-STAT6 fusion. This integrated cytology-ICC-histopathology approach not only helps in distinguishing SFT from its close morphological mimics but also underscores the expanding role of FNAC with ancillary techniques in precision diagnostics. Importantly, establishing a preoperative diagnosis allows surgeons to plan a complete yet eye-sparing excision with negative margins, thereby minimising morbidity and reducing the risk of recurrence. Given the potential for late relapse reported in SFTs, accurate early diagnosis and appropriate surgical management remain central to achieving optimal long-term clinical outcomes in these unconventional clinical scenarios.
